# Percutaneous Modified Blalock–Taussig Shunt Closure in a Patient with Isolated Right Ventricular Hypoplasia

**DOI:** 10.3390/jcdd10110460

**Published:** 2023-11-15

**Authors:** Stasa Krasic, Ivan Dizdarevic, Lana Vranic, Dejan Nešić, Vladislav Vukomanovic

**Affiliations:** 1 Cardiology Department, Mother and Child Health Institute of Serbia, 11070 Belgrade, Serbia; stasakrasic5@gmail.com; 2 Cardiac Surgery Department, Mother and Child Health Institute of Serbia, 11070 Belgrade, Serbia; ivandizdarevic@gmail.com; 3 Anesthesiology Department, Mother and Child Health Institute of Serbia, 11070 Belgrade, Serbia; 4 Faculty of Medicine, University of Belgrade, 11000 Belgrade, Serbia; drdejannesic@yahoo.com; 5 Faculty of Medicine, Institute of Medical Physiology, University of Belgrade, Visegradska 26/II, RS, 11129 Belgrade, Serbia

**Keywords:** isolated right ventricular hypoplasia, MBTS, MBTS percutaneous closure, AVP2

## Abstract

Clinical presentation, course, and treatment for patients with isolated right ventricular (RV) hypoplasia (IRVH) depends on the degree of hypoplasia that is present—this is a spectrum from spontaneous maturation to Fontan circulation over time. An 8-month-old infant presented with IRVH; in the patient, a modified Blalock–Taussig (MBTS) shunt was closed percutaneously after spontaneous RV function recovery. A female newborn was diagnosed with differential cyanosis at birth. The echocardiography showed a hypertrophic RV with a small cavity, a right–left shunt on the atrial septal defect, an almost closed ductus arteriosus (DA), and a small tricuspid valve ring (Z-score-2) with mild regurgitation (pressure gradient 30 mmHg). On the 4th day of life, the patient showed deepened cyanosis and hyperlactatemia was registered. The echocardiography examination revealed a closed DA. Right ventriculography performed on the 5th day of life evidenced the presence of a small hypertrabeculated RV. The pressure in the RV increased. A right-side MBTS was created on the 6th day of life. Further echocardiographic findings indicated a gradual development of the RV and a decrease in RV pressure. MBTS occlusion was performed when the patient was 8 months old. Vital parameters were monitored invasively and noninvasively after the balloon occlusion of MBTS. Percutaneous MBTS occlusion was successfully performed using an Amplatzer vascular plug 2 (AVP2). During the follow-up period, the patient was found to have maintained a normal percutaneous oxyhaemoglobin blood saturation.

## 1. Introduction

Isolated right ventricular (RV) hypoplasia (IRVH) was first described in 1950 by Cooley et al. [1]. IRVH is characterized by an underdeveloped RV with a small cavity, without severe pulmonary or tricuspid valvar malformations or ventricular septal defects (VSDs) [2]. This congenital heart disease results from a trabecular sinus portion development failure which is absent or marked as attenuated [3]. Due to IRVH, the tricuspid valve may be small or obstructive [2,3]. An associated atrial septal defect (ASD) or a patent foramen ovale (PFO) could persist and represent an escape valve, resulting in cyanosis. The clinical presentation depends on the degree of RV hypoplasia, interatrial communication size, and persistent pulmonary hypertension, and it has a broad outcome spectrum, from death in early infancy to mild cyanosis [2,3,4].

The natural history of this abnormality Is mostly relatively benign, with spontaneous RV and pulmonary circulation recovery. On the other hand, the data in the literature highlight the necessity of surgical treatment—ASD closure, systemic–pulmonary shunt (SPS), Glenn anastomosis, one-and-a-half repair, and Fontan circulation [2,3,4,5,6].

A female infant presented with IRVH; SPS was performed in the neonatal period and then they were percutaneously occluded at eight months of age with an Amplatzer vascular plug 2 (AVP2) after RV function recovery.

## 2. Case Report

**Figure 1:**
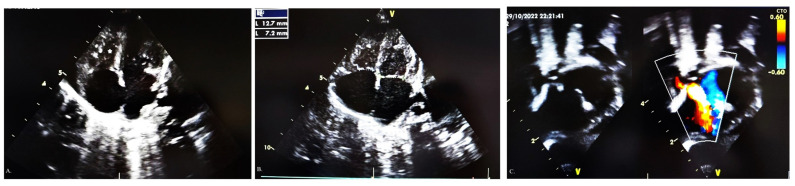
Echocardiographic finding in our patient at the admission: (**A**) small underdeveloped right ventricle (asterisk); (**B**) mild hypoplastic tricuspid valve (blue dotted line), 7.2 mm in diameter; (**C**) right–left shunt on atrial septal defect.

A female full-term newborn, born via spontaneous delivery weighing 2570 g, was diagnosed with differential cyanosis at birth. The mother smoked during pregnancy. The hyperoxia test was negative, so the suspicion of a congenital heart defect (CHD) was made, and she was transferred to a tertiary referral heart centre. On admission, transcutaneous oxyhaemoglobin blood saturation was 64%. ECG showed right atrial enlargement. The echocardiography displayed an undeveloped RV with a small cavity, right–left shunt on ASD, an almost closed DA, a small tricuspid valve ring (Z-score—2), and mild tricuspid valve regurgitation (pressure gradient 30 mmHg) (Figure 1). Prostaglandin E_2_ was initiated (50 ng/kg/min with graduated decreasing to 20 ng/kg/min). Immediately after, an apnoeic desaturation crisis was registered, the patient was intubated, and mechanical ventilation was started. She became hypotensive and oliguric, so dopamine (5 mcg/kg/min) was added to her therapy.

**Figure 2:**
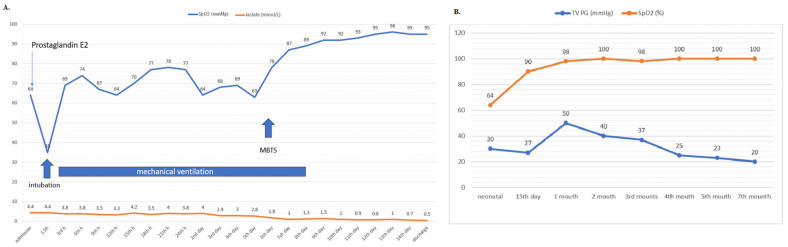
Dynamic transcutaneous oxyhaemoglobin blood saturation, tricuspid valve pressure gradient, and lactate during short- and middle-term follow-up. Abbreviations: SpO_2_—transcutaneous oxyhaemoglobin blood saturation; TV PG—tricuspid pressure gradient. (**A**) The graph shows the dynamics of changes in transcutaneous oxyhaemoglobin blood saturation and the lactate level during hospitalization (**B**) The graph shows the dynamics of gradient changes in transcutaneous oxyhaemoglobin blood saturation and on the tricuspid valve (estimated by echocardiography).

On the 4th day of life, the patient showed deepened cyanosis and hyperlactatemia was registered (Figure 2A). The echocardiography examination revealed a closed DA and a right–left shunt on the ASD.

**Figure 3:**
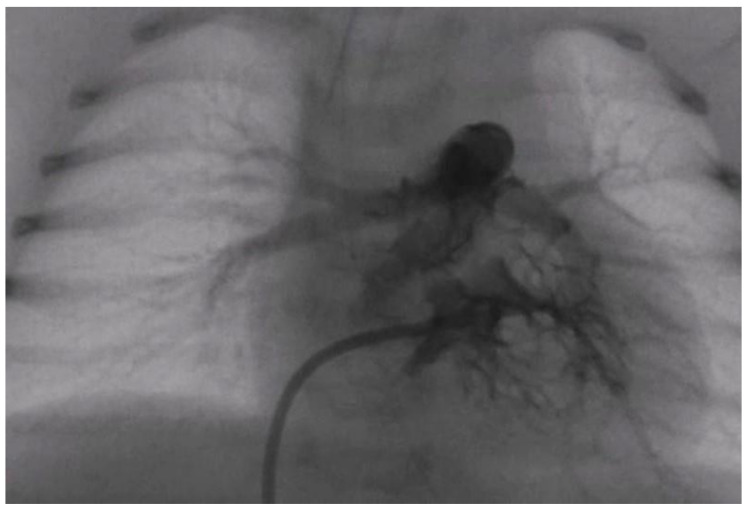
Postero–anterior view of a right ventricular angiography with opacification of the hypoplastic right ventricular cavum and the pulmonary artery with extensive contrast-free hypertrabeculation.

At the first cardiac catheterization on the 5th day of life, right ventriculography showed a small undeveloped RV with a well-developed pulmonary artery (PA) (Figure 3). Manometric tests indicated increased pressure in the RV (invasive pressure 46/6/16 mmHg) and PA (invasive pressure 34/13/21 mmHg), at an invasive systemic TA of 49/30/39 (Table 1). Frequent desaturation crises were registered during the catheterization and treatment with fentanyl and MgSO_4_ was administered.

As pulmonary hypertension was registered during the catheterization, inhalated NO was shortly used, while clinical improvement was not recorded. According to clinical presentation and feathers isolated right ventricle hypoplasia, palliative surgical intervention was indicated. On the 6th day of life, a right-sided modified Blalock–Taussig shunt (MBTS) of 3.5 mm was formed. The patient was discharged on the 16th day of life.

**Figure 4:**
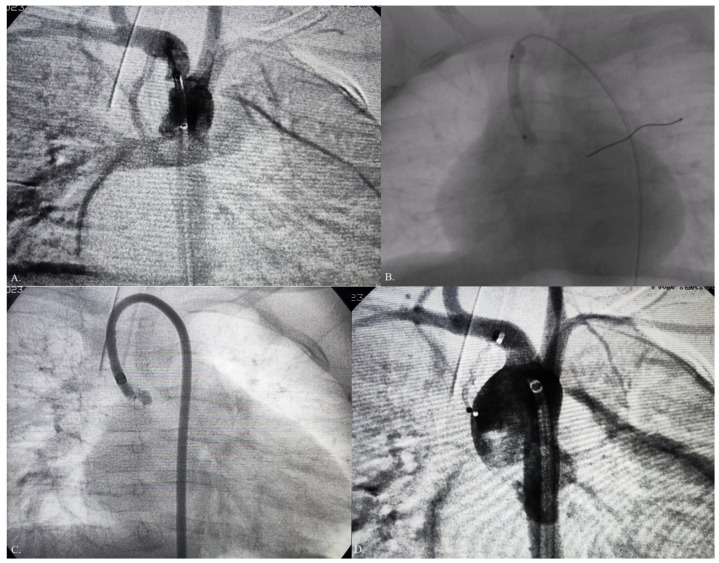
Modified Blalock–Taussig shunt (MBTS) occlusion using Amplatzer vascular plug 2 with the previous balloon test occlusion: (**A**) right oblique anterior 20° view of MBTS; (**B**) balloon occlusion MBTS test using a 4 × 20 mm TayShack balloon; (**C**) 5F J guiding catheter placed into MBTS; (**D**) aortography in postero–anterior view after MBTS occlusion with AVP2.

Transcutaneous oxyhaemoglobin blood saturation (SpO_2_ 100%) increased gradually during the short-term follow-up. Echocardiographic findings indicated a gradual decrease in the right ventricle pressure (Figure 2B) and an increase in the RV cavity with moderate tricuspid regurgitation. The patient was referred for transcatheter closure of an MBTS at 6 months. Heart catheterization was performed with the MBTS occlusion test using a 4 × 20 mm TayShack balloon (Figure 4A,B), and vital parameters were monitored (Table 1). Transcutaneous oxyhaemoglobin saturation remained at 100%, while the SaO_2_ of the blood in the RV was 66%. A 4F pigtail catheter was placed into the ascending aorta through the right femoral artery. Aortography in the AP and RAO 20° position showed a long tortuous MBTS that was 3.5 mm in diameter. A 4F Judkins catheter was placed in the MBTS and PA. Through 4F coronary catheter, an exchange length of 0.035-inch stiff guidewire (Amplatz super stiff wire, Boston Scientific, West Zone, Singapore) was placed into the left pulmonary artery. MBTS was occluded with the AVP2 5 mm by using a 5F J guiding catheter. Control aortography did not register the residual flow (Figure 4C,D). In further clinical course, normal transcutaneous oxyhaemoglobin blood saturation was maintained. The echocardiographic findings indicated a left–right shunt on the ASD and sufficient blood flow across the right ventricle inlet and outlet tracks without residual MBTS flow. X-ray findings evidenced good device positioning. The patient was discharged from the hospital after 5 days, with normal colour Doppler flows on both legs. An echocardiography examination 6 months after discharge revealed mild tricuspid regurgitation and a well-developed RV with normal RV pressure.

## 3. Discussion

Isolated right ventricle hypoplasia is a rare anomaly that is characterized by the underdevelopment of the trabecular portion alongside typically developed pulmonary and tricuspid valves. The etiology, clinical presentation, natural history, and treatment recommendations are based only on case reports in the literature. IRVH may be a primary developmental anomaly or may be due to a reduced tricuspid flow during foetal life. Still, some authors believe premature closure of the DA in utero or within 24 h after delivery may be another rare cause of IRVH. DA was almost closed in our patient 12 h after delivery. Like most literature cases, our patient had deep cyanosis and elevated blood lactate levels after birth [2].

Treatment options range from medical therapy to Fontan surgery, and in this case, the recorded RV dimensions were the main criteria for choosing the surgical method [4,5]. The majority of patients recorded in cases in the literature underwent ASD closure, but those patients had arterial oxygen saturation compared to patients who underwent Glenn operation or one-and-a-half ventricular repair [2]. On the 4th day of life, our patient had clinical worsening with transcutaneous oxygen blood saturation decreasing and increasing blood lactate levels while she underwent surgery.

The gradual recovery of the RV lumen among patients with IRVH has been previously described. Lombardi et al. presented three newborns with spontaneous IRVH resolution during infancy; here, transcutaneous oxygen blood saturation was 88% in two cases and 70% in one. The patients’ clinical statuses improved on oxygen administration; only one was referred for surgery to create an MBTS, but within 5 days, arterial oxygenation improved, and surgery was no longer necessary [3]. Our patient had a progressive decrease in transcutaneous oxygen blood saturation and hyperlactatemia with frequent pulmonary hypertension crises without improvement during the first days of life; therefore, she was referred to palliative surgery.

The gradual increase in the RV cavity and the normalization of cardiac size, RV, and pulmonary function were achieved for our patient after several months. We decided to occlude MBTS percutaneously to avoid surgery.

An MBT is usually clamped during Glenn or Fontan surgery, while transcatheter closures have been restricted to overflowing (pulmonary atresia with intact ventricular septum (AAP/IVS) or critical pulmonary stenosis (PS) who have previously undergone a decompressive surgical or interventional procedure) and residual shunts (Class I, Level of Evidence: C) [7]. Surgical closure of BT shunts is usually associated with an extended hospital stay, with high risk due to sternum re-incision, and a need for blood transfusions, with nerve and thoracic duct injury. At the same time, percutaneous closure is not a routine procedure; while it is technically challenging, it carries a higher rate of device embolization to the pulmonary artery [8,9,10,11,12]. Perry et al. occluded fourteen BTSs; in three patients, embolization of the overflowing shunt after surgical correction of AAP/IVS and PS eliminated the need for further surgery [10]; meanwhile, Sivakumar et al. performed a hybrid approach to occlude BTS before the surgical correction of Tetralogy of Fallot [11]. After spontaneous RV development, we performed transcatheter occlusion of the overflowing MBTS in an 8-month-old infant with IRVH.

Percutaneous occlusion has been performed electively using different techniques employing various types of coils and devices, including coils, detachable balloons, the Rashkind double-umbrella devices, Gianturco–Grifka vascular occlusion devices, duct occluders, and vascular plugs [8,9,10,11,12]. While coils occlusion has shown a significant incidence of migration (especially where there is no stenosis in the shunt) and usually needs distal balloon occlusion to prevent embolization or the use of bioptome cup forceps, we decided to use AVP2, which was ~50% larger in diameter than the target vessel diameter [8,9,10,11,12]. Additionally, we thought about AVP4 or Piccolo due to the possibility of using 4F guide catheter, but we did not have those devices available. Additionally, we use the 5F guide catheter to avoid femoral artery damage. Additionally, larger sheaths may kink because of acute angles created by sharp angles at the take-off and the insertion of surgically created shunts [7]. Consequently, Jang et al. placed AVP 6 mm in 3.5–4 mm MBTS with the catheter–snare technique (Table 2) [9]. We had problems reaching the MBTS with the guide catheter, but we finally succeeded without using the catheter–snare technique.

## 4. Conclusions

Isolated RVH is a rare cyanogenic congenital heart defect, which occasionally requires palliative surgical treatment, depending on the lumen of the RV. Transient and reversible IRVH has been previously described, and according to our case, it is also possible after MBTS creation. MBTS occlusion using AVP2 is a safe and feasible procedure in such patients, even in infancy, as it prevents surgical ligation of the MBTS with subsequent complications. This is the first patient in whom percutaneous MBTS occlusion was performed in infancy due to reversible IRVH.

## Figures and Tables

**Table 1:** Invasive manometric and oximetric measurements.

	Cath 1st		Cath 2nd		
	Pressure (mmHg)	SaO_2_ (%)	Pressure (mmHg)	SaO_2_ (%)	
				Before	With
Left atrium	14/2/7	64			
Left chamber	55/0/8				
AoAsc	49/25/31	66	97/52/73	100	100
AoDsc	48/30/37	66			
Right atrium	11/4/7	72.5			
SVC	9/4/7	59			
Right chamber	40/3/13	72	26/0/4	72	72
Pulmonary artery	34/13/21		23/10/15	88.6	66

Abbreviations: Cath—cardiac catheterization; AoAsc—ascending aorta; AoDsc—descending aorta; SVC—superior vena cava; SaO_2_—oxygen saturation.

**Table 2:** Literature review of percutaneous occlusion of Blalock–Taussig shunts.

Authors	Year	Number of Patients	Years of Age (Min–Max)	Congenital Heart Disease	Operation	Indication for MBTS Closure	Devices	Outcome
Agrawal [13]	2023	1	23	ToF	BTS; complete correction.	Residual shunt	AVP2	Successful
Surmacz [14]	2012	1	4	D-TGA, VSD, PS.	MBTS, complete correction (spontaneous MBTS occlusion).	Residual shunt	Coils (brachial artery access)	Successful
Rios-Méndez RE [8]	2009	3	1–23	L-TGA, HLHS, ASD, subvalvular PS; AAP/IVS; Single ventricle with a double entrance.	Bilater MBTS; right Glenn anastomosis; Right MBTS; surgical valvulotomy; Right MBTS; left Glenn; Fontan.	Residual left MBTS; Overflowing shunt; Residual right MBTS.	AVP (in one-patient coils)	Successful (embolization of coils)
Jang [9]	2008	1	1.5	Critical PS and RV hypoplasia.	Balloon valvuloplasty; right ventricular outflow tract reconstruction, right MBTS.	Overflowing shunt	AVP (snare technique)	Successful
Ramakrishnan [12]	2008	1	4	DORV, PS, D-TGA, situs inversus.	Right MBTS; left Glenn shunt and MBTS clipping.	Residual shunt	AVP (left jugular venous access)	Successful
Sivakumar [11]	2007	22	1–13 years (median age 4 years)	ToF	MBTS	Immediately before total surgical correction of ToF	16—coils (in 6 bioptome controlled; 3—proximal/distal flow occlusion); 6—AVP	13/16 successful (2 were occluded with ADO, 1 surgically); 6/6 successful
Kenny [15]	2007	1	57	ToF	BTS; complete correction.	residual shunt	ADO II	Successful
Benito [16]	2003	1	7	L-TGA, AAP/IVS, RV hypoplasia	Left MBTS; Glenn; Fontan.	non-reachable at the operation	Amplatzer ductal device (axillary artery access)	Successful
Limsuwan [17]	2000	1	6	ToF	Left MBTS, complete correction.	Residual shunt	Colis (snare technique and distal flow occlusion)	Successful (1 embolized)
Hoyer [18]	1999	1	15	L-TGA, pm VSD, PS, mild Ebstein’s anomaly, complete heart block.	Right BTS, permanent pacemaker; left MBTS; complete repair.	Residual left MBTS	Gianturco–Grifka vascular occlusion device (snare technique)	Successful
Tometzki [19]	1995	2	1.5–3.5	Critical PS	Open pulmonary valvotomy and MBTS; Balloon dilatation and MBTS.	Overflowing shunts	PFM duct occlusion (distal flow occlusion with a balloon)	Successful
Burrows [20]	1993	18	7 months—14.5 years (mean age of 6.2 years)	12—RV hypoplasia with AAP or PS; 4—complex congenital heart defects; 2—ToF.	12—right ventricular outflow tract reconstruction; 4—Glenn shunt or Fontan; 2—complete repair and bilateral BTS.		13—coils; 2—detachable balloons; 2—DO.	4/13—pulmonary embolism; 1/2—pulmonary embolism.
Houde [21]	1993	3	4–6	TA, D-TGA, hypoplastic RV, VSD, PS; TA, D-TGA, hypoplastic RV, VSD, AAP; AAP/IVS.	MBTS; VSD enlargement; Fontan; Bilateral MBTS; bilateral Glenn and right MBTS ligation; MBTS and trans-arterial pulmonary valvotomy; complete repair (failed); BH and Glenn shunt.	Overflowing	Rashkind occluding devices (in one patient, coil).	Successful (1 coil embolization)
Perry [10]	1989	14		5 ToF (2 with AAP); 1 TA; 3 AAP/IVS; 2 PS; 1 Fontan; 1 DORV/CAVC/PS; 1 D-TGA, VSD, AAP.		5—residual shunts; 9—overflowing shunts.	Coils (1–5 devices)	6—successful; 5—subtotal; 2—partial; 1—unsuccessful.
Reidy [22]	1983	1	11	ToF	BTS; complete correction	Residual shunt	Silicone-filled balloon	Successful
Culham [23]	1981	1	4	TA	Left BTS; right BTS	Overflowing shunt	Coils	Successful

Abbreviations: AAP—pulmonary artery atresia; ADO—Amplatzer ductal occlude; AVP—Amplatzer vascular plug; ASD—Atrial septal defect; BH—Blalock–Hanlon; BTS—Blalock–Taussig shunt; CAVC—complete atrioventricular canal; DO—ductal occlude; DORV—double outlet right ventricle; D-TGA—D transposition of the great vessels; HLHS—hypoplastic left heart syndrome; IVS—intact ventricular septum; L-TGA—L transposition of the great vessels; MBTS—modified Blalock–Taussig shunt; PS—pulmonary stenosis; RV—right ventricle; VSD—ventricular septal defect; TA—tricuspid atresia; ToF—tetralogy of Fallot.

## Data Availability

Data available on request due to restrictions eg privacy or ethical.

## References

[1] Cooley R.N., Sloan R.D., Hanlon C.R., Bahnson H.T. (1950). Angiocardiography in congenital heart disease of cyanotic type. II observations on tricuspid stenosis or atresia with hypoplasia of the right ventricle. Radiology.

[2] Hirono K., Origasa H., Tsuboi K., Takarada S., Oguri M., Okabe M., Miyao N., Nakaoka H., Ibuki K., Ozawa S. (2022). Clinical Status and Outcome of Isolated Right Ventricular Hypoplasia: A Systematic Review and Pooled Analysis of Case Reports. Front. Pediatr..

[3] Lombardi M., Tagliente M.R., Pirolo T., Massari E., Vairo U. (2016). Transient and anatomic isolated right-ventricular hypoplasia. J. Cardiovasc. Med..

[4] Cinteză E.E., Nicolescu A.M., Iancu M.A., Ganea G., Dumitru M., Dumitra G.G. (2022). Isolated hypoplastic right ventricle—A challenge in medical practice. Rom. J. Morphol. Embryol..

[5] Khajali Z., Arabian M., Aliramezany M. (2020). Best management in isolated right ventricular hypoplasia with septal defects in adults. J. Cardiovasc. Thorac. Res..

[6] Gewillig M., Brown S.C., De Catte L., Debeer A., Eyskens B., Cossey V., Van Schoubroeck D., Van Hole C., Devlieger R. (2009). Premature foetal closure of the arterial duct: Clinical presentations and outcome. Eur. Hear. J..

[7] Feltes T.F., Bacha E., Beekman R.H., Cheatham J.P., Feinstein J.A., Gomes A.S., Hijazi Z.M., Ing F.F., de Moor M., Morrow W.R. (2011). Indications for cardiac catheterization and intervention in pediatric cardiac disease: A scientific statement from the American Heart Association. Circulation.

[8] Rios-Méndez R.E., Gamboa R., Mollón F.P. (2009). Percutaneous closure of a modified Blalock-Taussig shunt using an amplatzer vascular plug. Rev. Esp. Cardiol..

[9] Jang G.Y., Son C.S., Lee J.W. (2008). Transcatheter occlusion of a modified Blalock–Taussig shunt using the amplatzer vascular plug with the catheter–snare technique. Pediatr. Cardiol..

[10] Perry S.B., Radtke W., Fellows K.E., Keane J.F., Lock J.E. (1989). Coil embolization to occlude aortopulmonary collateral vessels and shunts in patients with congenital heart disease. J. Am. Coll. Cardiol..

[11] Sivakumar K., Krishnan P., Pieris R., Francis E. (2007). Hybrid approach to surgical correction of tetralogy of Fallot in all patients with functioning Blalock Taussig shunts. Catheter. Cardiovasc. Interv..

[12] Ramakrishnan S., Kothari S.S. (2008). Amplatzer vascular plug closure of a Blalock–Taussig shunt through a Glenn shunt. Catheter. Cardiovasc. Interv..

[13] Agrawal V., Garg P., Vyas P., Hasit J., Mishra A. (2023). Hybrid approach for postclassical blalock–Taussig shunt tetralogy. J. Pract. Cardiovasc. Sci..

[14] Surmacz R., Moszura T., Mroziński B., Baszko A., Wojtalik M., Bobkowski W., Siwińska A. (2012). Transcatheter closure of Blalock-Taussig anastomosis with brachial access in a child with obstruction of the left subclavian artery and secondary subclavian steal syndrome. Adv. Interv. Cardiol. Postępy Kardiol. Interwencyjnej.

[15] Kenny D., Walsh K.P. (2008). Transcatheter occlusion of a classical BT shunt with the Amplatzer Duct Occluder II. Catheter. Cardiovasc. Interv..

[16] Bartolomé F.B., Martínez F.P., Fernández-Bernal C.S. (2003). Closure of a Blalock-Taussig shunt with an Amplatzer sevice after the Fontan operation (Cierre de la fístula de Blalock-Taussig con dispositivo de Amplatzer tras la operación de Fontan). Rev. Esp. Cardiol..

[17] Limsuwan A., Sklansky M.S., Kashani I.A., Shaughnessy R.D., Lucas V.W., Rothman A. (2000). Wire-snare technique with distal flow control for coil occlusion of a modified Blalock-Taussig shunt. Catheter. Cardiovasc. Interv..

[18] Hoyer M.H., Leon R.A., Fricker F.J. (1999). Transcatheter closure of modified Blalock-Taussig shunt with Gianturco-Grifka Vascular Occlusion Device. Catheter. Cardiovasc. Interv..

[19] Tometzki A.J., Houston A.B., Redington A.N., Rigby M.L., Redel D.A., Wilson N. (1995). Closure of Blalock-Taussig shunts using a new detachable coil device. Br. Heart J..

[20] Burrows P.E., Edwards T.C., Benson L.N. (1993). Transcatheter occlusion of Blalock-Taussig shunts: Technical options. J. Vasc. Interv. Radiol..

[21] Houde C., Zahn E.M., Benson L.N. (1993). Transcatheter closure of Blalock-Taussig shunts with a modified Rashkind umbrella delivery system. Br. Heart J..

[22] Reidy J.F., Baker E., Tynan M. (1983). Transcatheter occlusion of a Blalock-Taussig shunt with a detachable balloon in a child. Br. Heart J..

[23] Culham J., Izukawa T., Burns J., Freedom R. (1981). Embolization of a Blalock-Taussig shunt in a child. AJR Am. J..

